# The Prevalence of High-Risk HPV Types and Factors Determining Infection in Female Colombian Adolescents

**DOI:** 10.1371/journal.pone.0166502

**Published:** 2016-11-15

**Authors:** Luisa Del Río-Ospina, Sara Cecilia Soto-De León, Milena Camargo, Ricardo Sánchez, Cindy Lizeth Mancilla, Manuel Elkin Patarroyo, Manuel Alfonso Patarroyo

**Affiliations:** 1 Molecular Biology and Immunology Department, Fundación Instituto de Inmunología de Colombia (FIDIC), Bogotá D.C., Colombia; 2 School of Medicine, Universidad Nacional de Colombia, Bogotá D.C., Colombia; 3 Universidad de Ciencias Aplicadas y Ambientales (UDCA), Bogotá D.C., Colombia; 4 PhD Programme in Biomedical and Biological Sciences, Universidad del Rosario, Bogotá D.C., Colombia; 5 School of Medicine and Health Sciences, Universidad del Rosario, Bogotá D.C., Colombia; Universidad de Chile, CHILE

## Abstract

This study reports six HR-HPV types’ infection prevalence discriminated by species and multiple infection in unvaccinated Colombian female adolescents, as well as some factors modulating the risk of infection. HPV DNA for six high-risk viral types was identified in cervical samples taken from 2,134 12–19 year-old females using conventional generic and type-specific PCR. Binomial logistical regression analysis was used for modelling HR-HPV infection and multiple infection risk. The interaction between variables in a stepwise model was also included in such analysis. Viral DNA was detected in 48.97% of the females; 28.52% of them had multiple infections, HPV-16 being the most frequently occurring type (37.44%). Cytological abnormality prevalence was 15.61%. Being over 16 years-old (1.66: 1.01–2.71 95%CI), white ethnicity (4.40: 1.16–16.73 95%CI), having had 3 or more sexual partners (1.77: 1.11–2.81 95%CI) and prior sexually-transmitted infections (STI) (1.65: 1.17–2.32 95%CI) were associated with a greater risk of HPV infection. Having given birth was related to a higher risk of infection by A7 species and antecedent of abortion to less risk of coinfection. Where the females in this study came from also influenced the risk of infection by A7 species as female adolescents from the Andean region had a lower risk of infection (0.42: 0.18–0.99 95%CI). The presence of factors related to risky sexual behaviour in the study population indicated that public health services should pay special attention to female adolescents to modify the risk of infection by high-risk HPV types and decrease their impact on this age group.

## Introduction

The human papillomavirus (HPV) causes cervical cancer (CC), this being one of the five most frequently occurring types of cancer according to the International Agency for Research on Cancer (IARC). It is the second most frequently occurring type of cancer in Colombia, mainly affecting reproductive-aged females [1]. In addition to CC being caused/triggered by persistent infection by high-risk (HR) viral types, other factors modulate viral infection and the risk of CC [2]; many of such risk factors can be found in adolescents (casual sexual encounters, several sexual partners, early onset of sexual activity) thereby increasing the risk of viral infection. This means that they are considered an HR age-group [3]. Furthermore, diagnosis is difficult in adolescents, as is recording cases of sexually-transmitted infection (STI) (including the determination of HPV infection) due to poor adherence to promotion and prevention (P&P) programmes [3].

Variable HPV prevalence has been reported in young females [4–6]; however, most studies have dealt with different age ranges and few studies have evaluated viral distribution amongst female adolescents [7, 8]. Determining HPV prevalence and the occurrence of CC in this age group is thus not very accurate. It is known that CC has less impact on female adolescents than older women (15%) [9]; however, despite precursor lesions usually regressing in younger females, unnecessary procedures usually induce greater psychosexual morbidity and might have a negative impact on young females’ reproductive lives [10]. Females at risk of acquiring HPV, having persistent infections and/or developing CC must thus be identified (bearing the factors to which they have been exposed in mind). Studies are required which just focus on the adolescent population and which enable identifying HR-HPV types’ prevalence, as well as the variables which might increase or reduce the risk of CC; such females are the target population for HPV vaccination campaigns.

This study was aimed at estimating six HR-HPV types’ infection prevalence, as well as the sociodemographic and behavioural factors, which might be associated in the target, risk group (i.e. female Colombian adolescents). No convincing data regarding Colombia is currently available for providing new means of detection or improving P&P programmes, treatment and/or the timely control of cervical abnormalities in female adolescents.

## Materials and Methods

### Study design and population

This was a cross-sectional study involving female Colombian adolescents (12–19 years-old) who were voluntarily attending CC P&P programmes provided by the health services of 19 municipalities/cities in Colombia between March 2007 and May 2012. The females included in the study represented four geographically distinct regions of Colombia (Caribbean, Pacific, Andean and South-eastern), having different characteristics regarding relief, climate, vegetation and soil. There were also cultural, economic and social differences. The Caribbean region was represented by females from Barranquilla (Atlántico department), Cartagena (Bolívar department), Guajira department and Santa Marta (Magdalena department); the Pacific region was represented by females from Quibdó (Chocó department), Jamundí (Valle del Cauca department), Popayán (Cauca department), Tumaco and Ipiales (Nariño department). Bogotá (the capital of Colombia), Soacha, Girardot and Guaduas (Cundinamarca department), Apartadó (Antioquia department), Bucaramanga (Santander department) and Chaparral (Tolima department) are part of the Andean region, whilst Villavicencio (Meta department), Leticia (Amazonas department) and Mocoa (Putumayo department) provided females from the southern region.

The females in the study agreed to answer a questionnaire concerning sociodemographic data, sexual behaviour, smoking, family planning method, parity (# of deliveries > 20 weeks) and having a background of STI. A cervical sample was then taken which was used for the Papanicolaou test (Pap test) and for the molecular detection of HPV. Pap test results were reported according to the Bethesda system; females having an abnormal result had a colposcopy [11].

### Detecting and typing HPV by PCR

The methodology used for viral extraction, detection and typing has already been reported [12, 13]; cytobrushes were used for taking cervical samples (i.e. the Pap test) and preserved in medium with 95% ethanol at 4°C until being processed. Sample quality was verified; the presence of DNA was confirmed by conventional PCR amplification of the *β-globin* gene with GH20/PC04 primers [14]. Three sets of primers were used (GP5+/GP6+, MY09/MY11 and pU1M/2R); they were directed towards the viral genome’s L1 and E6/E7 regions for determining the presence of HPV. These primers enabled identifying infections having low viral loads and the detection of coinfection, thereby ensuring greater test robustness and sensitivity [14, 15]. The PCR conditions and protocols for each primer have been reported previously [12]. HR-HPV types 16, 18, 31, 33, 45 and 58 were typed in samples proving positive by PCR by at least one set of generic primers [12]. Positive and negative controls were used in all PCR assays to verify whether there was contamination and detect unexpected results (the corresponding assay was repeated if this was the case). PCR results were visualised by electrophoresis on 2% agarose gel and stained with SYBR safe (Invitrogen, CA, USA) [12].

### Ethical considerations

Prior to the start of the study, a psychological evaluation verified minors’ understanding, reasoning and logical capability (Colombian guidelines / resolution 008430/1993) [16]. Females who accepted the invitation to participate signed an informed consent form whilst supervised by their tutors (if they were minors) or a witness. The present study’s protocol and procedures were approved and supervised by all the ethics committees of the healthcare entities participating in the study: Comité de Ética en Investigación en el Área de la Salud de la Universidad del Norte (Barranquilla), E.S.E Clínica Maternidad Rafael Calvo (Cartagena), E.S.E Hospital San Rafael Nivel II (Guajira), E.S.E. Hospital Alejandro Próspero Reverendo (Santa Marta), E.S.E Hospital Local Ismael Roldán Valencia (Quibdó), Hospital Piloto (Jamundí), Hospital Nivel II Susana López de Valencia (Popayán), E.S.E. Hospital San Andrés (Tumaco), Centro de Salud Nuestra Señora del Pilar E.S.E. and IPS de los Cabildos Indígenas del Gran Cumbal, Panán, Chiles y Mayasquer (Ipiales), Hospital de Fontibón E.S.E, Hospital de Engativá (Nivel II) and Hospital de Bosa–E.S.E (Nivel II) (Bogotá), E.S.E. Hospital Mario Gaitán Yaguas (Soacha), Hospital San Rafael (Girardot), IPS Hospital San José de Guaduas, E.S.E. Hospital Antonio Roldán Betancur (Apartadó), E.S.E. Instituto de Salud–ISABU (Bucaramanga), Hospital San Juan Bautista (Chaparral), Liga Colombiana contra el Cáncer—Seccional Meta, Liga Contra el Cáncer–Seccional Leticia, E.S.E Hospital José María Hernández (Mocoa) and Fundación Instituto de Inmunología de Colombia (Bogotá).

### Statistical analysis

For analysis purposes, multiple infections were taken as the presence of at least one viral type. Percentages were reported for categorical variables, along with their respective 95% confidence intervals (95%CI); medians with interquartile ranges (IQR) were used for continuous variables (i.e. non-normal data). Bivariate analysis was used for finding differences in percentages for females having or without HPV infection, i.e. χ^2^ test or Fisher’s exact test depending on the values observed in each category.

Cervical lesion frequency was analysed regarding age, as was multiple infection frequency, infection by specific type regarding the degree of lesion reported in cytology (p trend values were reported).

Binomial logistical regression analysis was used for modelling the risk of HR-HPV infection, infection by A9 and A7 species and infection by more than one viral type. Crude and adjusted odds ratios (OR) were reported, together with 95%CI. The models were adjusted for age, region, ethnicity, monthly income, civil state, the amount of sexual partners, STI incidence, a background of births and /or previous abortions, contraceptive method used and whether the participant smoked. A stepwise model was used for assessing association and interaction between variables (0.15 as p.e and 0.2 as p.r).

Stata 12 software was used for all statistical analysis (0.05 significance level).

## Results

This study initially involved 2,253 12 to 19 year-old females; females were excluded if the control gene (*β-globin*) could not be amplified (n = 123; 5.46%) in their samples. Generic viral identification, followed by type-specific identification, was used on the remaining 2,134 females. It was found that 1,045 young females were positive for HPV (48.97%: 46.83–51.11 95%CI); 70.62% (n = 738: 67.75–73.37 95%CI) of HPV positive females had single infections whilst 28.52% (n = 298: 25.79–31.36 95%CI) were positive for more than one HR viral type.

The most frequently occurring viral type in this group of females was HPV-16 (37.44%: 35.38–39.53 95%CI), followed by HPV-18 (14.99%: 13.50–16.58 95%CI). The infecting viral type was not established in 9 HPV positive females (0.86%) since they were negative for the types identified in this study (HPV-16, -18, -31, -33, -45 and -58), suggesting another HPV type.

**Fig 1:**
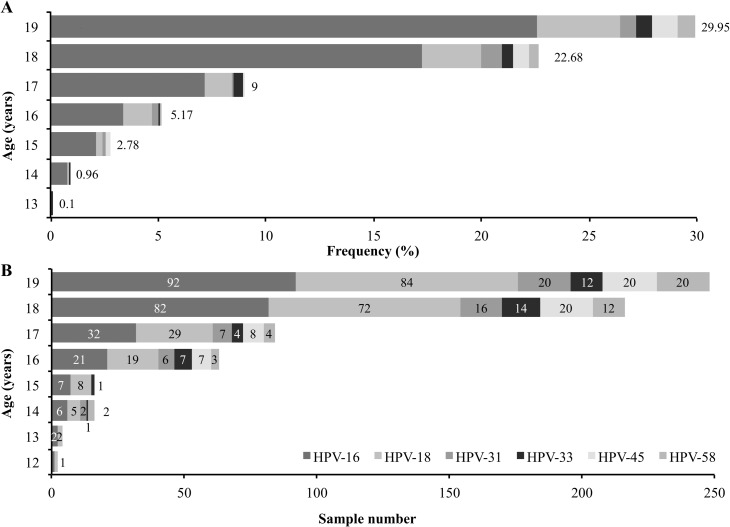
**High-risk viral type distribution by age: A.** Single infection frequency; **B**. The number of positive samples regarding multiple infections.

Fig 1 shows type-specific distribution in single and multiple infections according to the age of the females in the sample. It was observed that the percentage of single infections increased with age, even though the same was not observed regarding multiple infections (Table 1, Fig 1). HPV-16 was the most frequently occurring viral type in single infections, followed by HPV-18 and HPV-31. Regarding coinfection other than HPV-16, HPV-45 was the second type concerning frequency of appearance.

**Table 1:** Sociodemographic characteristics and risk factors regarding HPV infection.

Variable	Overall	%	HPV negative (n = 1,089)		HPV positive ^a^				p ^c^
					Single (n = 738)		Multiple ^b^ (n = 298)		
			n	%	n	%	n	%	
**Age in years (n = 2,134)**									0.759
12	2	0.09	1	50.00	0	0.00	1	50.00	
13	10	0.47	7	70.00	1	10.00	2	20.00	
14	34	1.59	16	47.06	10	29.41	8	23.53	
15	80	3.75	43	53.75	29	36.25	8	10.00	
16	178	8.34	96	53.93	54	30.34	28	15.73	
17	297	13.92	160	53.87	94	31.65	39	13.13	
18	682	31.96	341	50.00	237	34.75	101	14.81	
19	851	39.88	425	49.94	313	36.78	111	13.04	
**Region (n = 2,134)**									0.004
Caribbean	194	9.09	91	46.91	72	37.11	29	14.95	
Pacific	444	20.81	260	58.56	133	29.95	47	10.59	
Andean	1,323	62.00	653	49.36	474	35.83	194	14.66	
South-eastern	173	8.11	85	49.13	59	34.10	28	16.18	
**Ethnicity (n = 2,051)**									0.042
White	37	1.80	12	32.43	18	48.65	7	18.92	
Indigenous	57	2.78	25	43.86	15	26.32	17	29.82	
Mestizo	1,743	84.98	913	52.38	590	33.85	234	13.43	
Black	214	10.43	103	48.13	81	37.85	28	13.08	
**Average monthly income (n = 1,656)** ^**d**^									0.021
None	85	5.13	32	37.65	41	48.24	11	12.94	
< minimum	1,223	73.85	650	53.15	399	32.62	171	13.98	
≥ minimum	348	21.01	178	51.15	124	35.63	44	12.64	
**Marital status (n = 2,099)**									0.246
Single	1,097	52.26	547	49.86	396	36.10	152	13.86	
Not single	1,002	47.74	525	52.40	328	32.73	142	14.17	
**Lifetime amount of sexual partners (n = 2,100)**									0.000
0–2	1,879	89.48	984	52.37	634	33.74	254	13.52	
≥ 3	221	10.52	85	38.46	95	42.99	40	18.10	
**STI (n = 2,024)** ^**e**^									0.000
No	1,348	66.60	729	54.08	433	32.12	182	13.50	
Yes	676	33.40	309	45.71	268	39.64	96	14.20	
**Births (n = 1,653)**									0.513
No	817	49.43	410	50.18	296	36.23	109	13.34	
Yes	836	50.57	433	51.79	269	32.18	130	15.55	
**Abortions (n = 1,533)**									0.466
No	1,301	84.87	656	50.42	447	34.36	192	14.76	
Yes	232	15.13	123	53.02	84	36.21	24	10.34	
**Contraceptive method used (n = 1,965)**									0.305
None	763	38.83	374	49.02	273	35.78	113	14.81	
Condon	288	14.66	155	53.82	95	32.99	38	13.19	
Hormonal	772	39.29	403	52.20	274	35.49	91	11.79	
IUD	142	7.23	79	55.63	40	28.17	21	14.79	
**Smoker (n = 1,249)**									0.422
No	1,046	83.75	532	50.86	365	34.89	148	14.15	
Yes	203	16.25	97	47.78	80	39.41	26	12.81	

^a^ Nine samples proved negative for the six HR-HPV types detected.

^b^ Positive for two or more HR-HPV types.

^c^ χ^2^ test or Fisher’s exact test for HPV negative and HPV positive women.

^d^ The minimum average monthly income (2016 rate) would be roughly US $300.

^e^ Including trichomoniasis, candida, chlamydia, herpes, syphilis, gonorrhoea and genital warts.

Table 1 shows the females’ sociodemographic characteristics and some risk factors for CC (bearing HPV infection status in mind). Most females came from the Andean region (62%: 59.90–64.06 95%CI) and the fewest (173 females) from the south-eastern region (Amazonia and Orinoquia). Differences were found regarding the region they came from; the Andean (50.49%: 47.76–53.22 95%CI) and Caribbean regions (52.06%: 44.79–59.27 95%CI) had the highest HPV infection percentage (and single infection predominance). Regarding ethnicity, 84.98% of the females (83.36–86.50 95%CI) stated that they were mestizos; only in females who stated that they were Indigenous (n = 57; 2.78%) was the percentage of multiple infections greater than the percentage of single infections (29.82%: 18.43–43.40 95%CI compared to 26.32%: 15.53–39.66 95%CI). Few of the females in this study (i.e. most attending state-provided P&P programmes) had monthly income equal to or greater than Colombia’s minimum wage (21.01%: 19.07–23.06 95%CI); a higher percentage of HPV infection was found in females lacking income (61.18%: 49.99–71.56 95%CI).

Mean age at the start of sexual relationships was 15 years-old (IQR 14; 16 years); no statistically significant differences were observed between females having and without HPV (Mann-Whitney U test, p = 0.767). Females stating that they had had 3 or more sexual partners had a greater percentage of infection compared to females having had 0 to 2 sexual partners (61.09%: 54.32–67.55 95%CI compared to 38.46%: 32.01–45.22 95%CI); this was a statistically significant difference and further statistically significant differences were found for females having and lacking a background of STI (p = 0.000).

**Fig 2:**
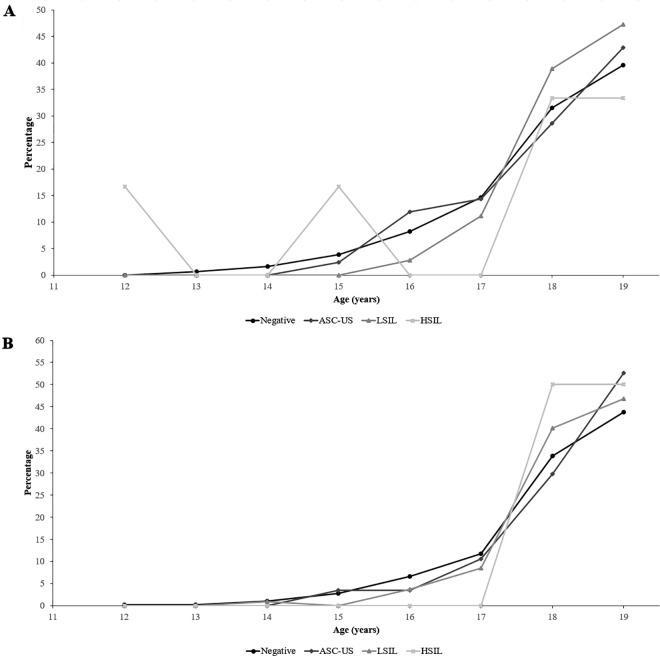
Cytological findings by age. **A.** HPV negative adolescents; **B.** HPV positive adolescents.

Some degree of cervical lesion was identified by Pap test in 250 females (15.61%: 13.86–17.48 95%CI). Atypical squamous cells of undetermined significance (ASC-US) were found in 39.60% of the females, low-grade squamous intraepithelial lesion (LSIL) in 57.20% and high-grade squamous intraepithelial lesion (HSIL) in 3.20%. Fig 2 shows cervical lesion frequency by age in HPV negative (A) and positive (B) females. Even though no lesions were identified in most adolescents, it was observed that lesion frequency increased with age (p trend = 0.004). An increase was also found in HR-HPV infection frequency, multiple infection and infection by specific species as the degree of lesion reported by cytology increased (Table 2).

**Table 2:** HPV infection distribution according to cytological diagnosis.

HPV infection	Overall (n = 1602)		Negative (n = 1357)		ASCUS (n = 99)		LSIL (n = 143)		HSIL (n = 8)		p trend
	n	%	n	%	n	%	n	%	n	%	
**HR-HPV** ^**a**^	772	47.65	606	44.66	57	57.58	107	74.83	2	25.00	0.000
**Single infection**	550	33.95	432	31.83	42	42.42	75	52.45	1	12.50	0.000
**Multiple infection** ^**b**^	216	13.33	169	12.45	14	14.14	32	22.38	1	12.50	0.003
**A9**	600	37.04	467	34.41	45	45.45	87	60.84	1	12.50	0.000
**A7**	275	16.98	225	16.58	17	17.17	33	23.08	0	0.00	0.163
**HPV-16**	600	37.04	467	34.41	45	45.45	87	60.84	1	12.50	0.000
**HPV-18**	227	14.01	188	13.85	14	14.14	25	17.48	0	0.00	0.464
**HPV-31**	52	3.21	39	2.87	4	4.04	8	5.59	1	12.50	0.004
**HPV-33**	42	2.59	29	2.14	1	1.01	12	8.39	0	0.00	0.000
**HPV-45**	60	3.70	46	3.39	3	3.03	11	7.69	0	0.00	0.006
**HPV-58**	47	2.90	36	2.65	4	4.04	6	4.20	1	12.50	0.023

^a^ HR-HPV infection was considered as positive for at least one primer used for detection by generic PCR.

^b^ Nine samples proved negative for 6 HR-HPV types detected.

The following variables were related to a greater risk of infection when using logistical regression analysis for modelling the risk of infection by HR-HPV types: age >16 years, white ethnicity, having had 3 or more sexual partners and a background of STI. When determining risk by species, the risk of infection by A9 (HPV-16, -31, -33 and -58) was associated with being over 16 years-old, as was a background of STI, whilst a background of births and STI conferred a risk of infection by A7 species (HPV-18 and -45). Factors such as previous abortions and coming from the Andean region appeared to be related to less risk of infection. When modelling the risk of coinfection in the study population, it was observed that births were associated with a greater risk and previous abortions with lower risk (Table 3).

**Table 3:** Factors associated with infection by HR-HPV types, by species and multiple infection.

Variable	HR-HPV						A9		A7				Multiple HPV ^a^			
	OR	95%IC	aOR	95%IC	OR	95%IC	aOR	95%IC	OR	95%IC	aOR	95%IC	OR	95%IC	aOR	95%IC
**Age**																
≤ 16 years	*Reference*				*Reference*				*Reference*				*Reference*			
> 16 years	1.13	0.88–1.44	**1.66**	**1.01–2.71**	1.22	0.94–1.57	**1.69**	**1.00–2.85**	0.86	0.63–1.17	1.03	0.52–2.03	0.87	0.62–1.23	0.82	0.40–1.67
**Region**																
Caribbean	**1.60**	**1.14–2.24**	1.24	0.29–5.33	**1.49**	**1.04–2.12**	1.81	0.42–7.87	1.36	0.89–2.07	2.71	0.59–12.52	1.49	0.90–2.45	3.91	0.74–20.54
Pacific	*Reference*				*Reference*				*Reference*				*Reference*			
Andean	**1.45**	**1.17–1.80**	0.87	0.46–1.64	**1.64**	**1.30–2.08**	1.59	0.80–3.14	1.05	0.79–1.40	**0.42**	**0.18–0.99**	**1.44**	**1.02–2.02**	0.79	0.30–2.09
South-eastern	**1.46**	**1.03–2.08**	0.94	0.36–2.47	**1.64**	**1.13–2.36**	1.94	0.72–5.25	1.29	0.83–2.01	0.47	0.11–1.98	1.62	0.98–2.69	0.96	0.21–4.28
**Ethnicity**																
White	**2.29**	**1.14–4.59**	**4.40**	**1.16–16.73**	1.16	0.60–2.26	1.24	0.41–3.78	**2.35**	**1.17–4.73**	2.22	0.64–7.75	1.50	0.65–3.45	0.51	0.06–4.10
Indigenous	1.41	0.83–2.39	0.77	0.25–2.33	1.43	0.84–2.43	0.96	0.29–3.17	**2.44**	**1.39–4.30**	1.39	0.38–5.05	**2.73**	**1.52–4.89**	1.87	0.46–7.59
Mestizo	*Reference*				*Reference*				*Reference*				*Reference*			
Black	1.18	0.89–1.57	1.40	0.72–2.72	1.08	0.81–1.45	1.84	0.93–3.66	1.16	0.81–1.67	0.96	0.40–2.30	0.98	0.64–1.49	1.46	0.57–3.77
**Average monthly income** ^**b**^																
None	**1.73**	**1.07–2.82**	1.54	0.59–4.04	1.20	0.74–1.94	0.74	0.27–2.03	1.35	0.73–2.51	1.68	0.48–5.83	1.03	0.51–2.10	1.63	0.46–5.72
< minimum	0.92	0.73–1.17	0.96	0.65–1.42	0.89	0.69–1.13	0.90	0.61–1.34	1.28	0.92–1.79	1.28	0.72–2.27	1.12	0.78–1.59	1.14	0.63–2.09
≥ minimum	*Reference*				*Reference*				*Reference*				*Reference*			
**Marital status**																
Single	*Reference*				*Reference*				*Reference*				*Reference*			
Not single	0.90	0.76–1.07	1.10	0.78–1.56	0.96	0.80–1.14	1.14	0.80–1.63	0.88	0.71–1.11	0.95	0.58–1.55	1.03	0.81–1.32	0.94	0.56–1.60
**Lifetime number of sexual partners**																
0–2	*Reference*				*Reference*				*Reference*				*Reference*			
≥ 3	**1.76**	**1.32–2.34**	**1.77**	**1.11–2.81**	**1.68**	**1.27–2.22**	1.50	0.95–2.37	1.29	0.92–1.81	1.38	0.75–2.52	1.41	0.98–2.04	1.76	0.95–3.26
**STI** ^**c**^																
No	*Reference*				*Reference*				*Reference*				*Reference*			
Yes	**1.40**	**1.16–1.68**	**1.65**	**1.17–2.32**	1.19	0.98–1.44	**1.44**	**1.02–2.03**	1.20	0.94–1.52	**2.09**	**1.31–3.32**	1.06	0.81–1.39	1.64	1.00–2.70
**Births**																
No	*Reference*				*Reference*				*Reference*				*Reference*			
Yes	0.94	0.77–1.14	1.04	0.72–1.49	0.99	0.81–1.20	0.94	0.65–1.37	1.06	0.82–1.36	**1.83**	**1.10–3.02**	1.20	0.91–1.58	**2.11**	**1.22–3.64**
**Abortions**																
No	*Reference*				*Reference*				*Reference*				*Reference*			
Yes	0.90	0.68–1.19	0.63	0.39–1.02	0.91	0.68–1.21	0.67	0.41–1.10	**0.64**	**0.43–0.97**	**0.29**	**0.12–0.72**	0.67	0.42–1.04	**0.23**	**0.08–0.67**
**Contraceptive method**																
None	1.21	0.92–1.59	1.27	0.78–2.08	1.22	0.92–1.62	1.21	0.73–2.00	1.16	0.81–1.65	1.35	0.66–2.77	1.15	0.77–1.71	0.74	0.36–1.51
Condon	*Reference*				*Reference*				*Reference*				*Reference*			
Hormonal	1.07	0.81–1.40	1.16	0.70–1.92	1.12	0.84–1.49	0.98	0.59–1.65	0.94	0.65–1.34	1.02	0.49–2.15	0.88	0.59–1.33	0.71	0.34–1.46
IUD	0.93	0.62–1.39	0.76	0.36–1.61	1.15	0.76–1.75	0.96	0.45–2.06	0.89	0.52–1.55	0.64	0.21–1.92	1.16	0.65–2.06	0.54	0.18–1.61
**Smoker**																
No	*Reference*				*Reference*				*Reference*				*Reference*			
Yes	1.13	0.84–1.53	1.08	0.69–1.68	1.10	0.81–1.49	1.00	0.63–1.58	0.94	0.63–1.42	1.64	0.91–2.96	0.89	0.57–1.39	1.60	0.86–2.96

Values in bold indicate statistical significance based on the 95% confidence interval, p < 0.05.

^a^ Positive for 2 or more HR-HPV types.

^b^ The minimum average monthly income (2016 rate) would be roughly US $300.

^c^ Including trichomoniasis, candida, chlamydia, herpes, syphilis, gonorrhoea and genital warts.

The stepwise model suggested that HR-HPV infection risk variables remained significant, including interaction terms for determining whether these had an effect on the model: being over 16 years-old (adjusted OR 1.64: 1.01–2.64 95%CI), white ethnicity (adjusted OR 3.95: 1.07–14.60 95%CI), ≥ 3 lifetime sexual partners (adjusted OR 1.75: 1.11–2.75 95%CI) and a background of STI (adjusted OR 1.60: 1.15–2.22 95%CI). Stepwise models were constructed considering the other outcomes (infection by species A9 and A7 and coinfection); it was found that the aforementioned variables maintained their association with outcomes (data not shown).

## Discussion

HPV prevalence was estimated in a female Colombian adolescent population and the factors which might have modulated the risk of infection were evaluated. It was found that age, ethnicity, the amount of sexual partners, a background of STI and previous births and abortions modulated the risk of HPV infection, infection by types from A9 and A7 species and infection by more than one viral type (Table 3).

Overall HPV infection prevalence varied according to geographical region, age and cervical abnormality [4–6]. Considering the study population’s age, the prevalence found (48.97%) was greater than that in previous reports regarding Colombian females in this age group (26.1% [17] 27.2% [5]); nevertheless, similar infection prevalence has been reported in a Latin-American population (44.2% [18]).

The females participating in the study came from 18 municipalities/cities outside Bogotá which have reported high infection rates [19]; these regions’ sociocultural and economic differences (mostly based on tourism) favour exposure to factors increasing the risk of acquiring HPV (risky sexual behaviour). The algorithm used for generic detection of HPV, with the 3 sets of primers, led to identifying infections having low viral loads and coinfection, thereby increasing the method’s robustness and facilitating the diagnosis of a greater amount of infections [15].

The highest rates of infection worldwide have been found in females under 25 years-old (up to 80%) [4, 5]; infection frequency in the present study increased regarding age from 0.10% in 12 year-old females up to 40.77% in 19 year-old females. HPV infection detection has a temporal relationship with the onset of engaging in sexual relationships, thereby explaining the first peak of detection in females aged less than 25 years-old [4]. Such prevalence has been seen to gradually decrease after 25 years-of-age (except in regions of Asia and Africa) and then increase towards 45 years-old in Latin-American and the Caribbean populations, known as left-skewed unimodal (Europe and North-America) and bimodal distribution [4, 5].

Worldwide 11%-12% HPV prevalence has been reported in females lacking cervical lesions; however, such prevalence increases proportionally with lesion severity (90% in females having cervical intraepithelial neoplasia (CIN3) and invasive cervical cancer (ICC)) [4, 6] (Table 2). As expected, a tendency was identified regarding increased infection related to greater lesion severity. The foregoing reflects results from previous studies reporting a greater risk of CIN3 and ICC following HPV infection [20]. Age plays an important role in determining the prevalence of cytological abnormalities. Cytological abnormality prevalence was 15.61%, lower than that found in <25 year-old females (20.89%-26.94%) [21, 22]. This difference was expected, considering that the females in this study were <19 years-old. Cervical lesion prevalence in this population increased as age increased, related to a greater risk of acquiring infection by HR-HPV types following the first sexual relationship in young females [7].

Regarding ethnicity, it was found that being white was associated with a greater risk of infection by HR-HPV types (67.57%: 50.21–81.99 95%CI); however, further studies are needed for determining these females’ ancestry and thus confirm such association. It was also found that this group of females was more likely to be exposed to HPV infection risk factors (hormonal planning method (51.31%) and a background of STI (45.94%)). Curiously, it was also determined that the region they came from influenced the risk of infection by types from the A7 species; females from the Andean region had a reduced risk. This might partly be explained by most of the population in this region being mestizo (91.83%: 90.23–93.25 95%CI) and the lowest A7 type infection percentage was found for this ethnicity (16.92%: 15.19–18.77 95%CI).

Previous studies have described the role of viral variants in CC, specifically depending on region and ethnicity [23, 24]. Possible mechanisms related to the immune response enable better elimination of infection by certain variants in specific populations [25, 26]. Evidence has been presented that genetic differences between racial groups might explain the functional diversity of immune response factors, inflammation, metabolism, etc., in response to HPV infection [27]. Further studies are thus needed for identifying HPV molecular variant prevalence in this age group and ethnicity to confirm such results.

Early onset of sexual activity has been related to a greater amount of sexual partners during adolescence and in young adults, thereby favouring acquiring other STI and, in some cases, pregnancy at an early age [3, 28, 29]. A greater number of sexual partners (≥ 3) has been associated with a greater risk of infection by HR-HPV types. Even though the sexual history of the sexual partners of the adolescents participating in the study was not evaluated, factors such as promiscuity, previous STI and their non-circumcision have been associated with greater circulation of viral types and thus multiple type transmission [30].

IARC studies in Lyon, France and by the Institut Català d’Oncologia (ICO) in Barcelona, Spain, have shown that the early onset of sexual relationships and pregnancies in adolescents increase the risk of developing CC due to early exposure to carcinogenic viral types, a longer period of adolescents’ susceptibility to HPV infection, the hormonal influence of steroids against HPV infection and host immune response during pre-adolescence and adolescence [31].

A mixture of columnar, squamous and metaplastic epithelium is found in the cervix of adolescents and young females; the squamous-columnar junction (transformation area where HPV infection tends to cause CC) is exposed, facilitating stratified epithelium base layer exposure to HPV regarding minimum trauma [31]. Hormonal influence becomes increased during adolescence and pregnancy, promoting squamous metaplastic transformation of the everted endocervical epithelium which (regarding HPV) increases the probability of malign transformation [32], mainly occurring during pregnancy at an early age [31].

Early onset of sexual relationships and pregnancy at an early age in females from developing countries usually occurs (as in this study) at around 15 years of age. Early maternity has been associated with a greater risk of CC, attributed to trauma during the first birth [31] as lesions are created in the birth canal and immunosuppression may even be induced in this area, thereby explaining an increased risk of acquiring the virus [33]. In the present study, a background of births was found to be related to a greater risk of infection by types from the A7 species and with infection by multiple HR-HPV types.

An inverse association between the risk of HPV infection and a background of abortion was identified, this having been previously reported in Mexican females [34]. Abortions occurring during the first trimester of pregnancy tend to involve less probability of causing a substantial increase in oestrogen levels, and trauma produced in spontaneous abortions is zero or minimal. The forgoing partly explains the reduced risk of infection by A7 species and by multiple types reported in the adolescents in the present study.

The relationship between STI and HPV infection is based on the inflammatory effects of other pathogens. It has been described that alterations and damage to cervical epithelium produced by pathogens such as *Chlamydia trachomatis*, *Trichomonas vaginalis* and *Candida* spp. promote cell proliferation, tissue debridement, free radical and cytosine release and facilitate colonisation, thereby improving microorganism virulence, increasing host susceptibility, facilitating HPV entry and reducing its elimination [35]. The present study found an association between previous STI and the risk of infection by HR-HPV types. It should be stressed that the adolescent population exposed to risky sexual behaviour should be screened for these infections which could participate in HPV persistence, redetection and tumour carcinogenesis through cumulative effects [36].

Studies have indicated that multiple infections favour the persistence of a single viral type, whether due to inter-genotype competition or a more effective type-specific immune response triggered by other viral types; this means that most cases of CC are caused by a single viral type [37]. The forgoing correlates with that found in this study, even though coinfection was only identified in 19.01% of the females having low grade lesions. Table 2 shows how the percentage of coinfection was greater regarding LSIL and lower in HSIL. Higher multiple infection prevalence has been found in young females, thereby agreeing with the relationship between greater sexual activity and the sexual transmission of multiple viral types [38]; other viral types not included in the vaccines available today have also been found in these cases (HPV-45). It was found in this adolescent population that previous births conferred a greater risk of confection and abortions a lesser risk, such findings being related to previous reports about the effect of hormonal influence, the presence of local lesions and immunosuppression concerning the natural history of the disease [32, 33].

A study dealing with an adolescent population provides relevant information for improving P&P and diagnosis programmes considering adolescents’ exposure to various factors such as those related to sexual behaviour, ethnicity and region of origin since they affect the risk of infection and coinfection.

These results suggested that the risk factors reported for HPV infection and developing CC could vary when discriminated by infecting species, viral type and multiple infections in an unvaccinated population such as the one studied here. This highlights the importance of having different aged populations during analysis for establishing individual risk and the influence of background and associated factors. The findings showed that adolescents’ risky sexual behaviour is a key factor modifying HPV infection dynamics. Encouraging delay regarding the start of sexual life would reduce the risk of HPV infection and also the risk of contracting other STI. The foregoing, together with other P&P strategies and the appropriate use of contraceptive methods [39] aimed at reducing pregnancy in adolescents could affect the burden represented by HPV infection in adolescents, the consequences and also the physical and emotional effects of the treatment required. Special attention should be given to regions having lower levels of education and socio-economic development as such conditions could be accompanied by less knowledge and understanding of the disease, thereby jeopardising the appropriate development of P&P programmes [3].

Despite young females having a lower relative risk of acquiring CC, there is a slight risk of non-participation. It has been demonstrated that a lack of opportunity for prevention increases risk by reducing this population’s commitment to self-care and participating in the healthcare programmes available in each region [39, 40]. Behavioural intervention strategies should be promoted; others should promote the diffusion of knowledge for adolescents and encourage their participation in screening programmes aimed at identifying HPV infected females exposed to determinants modifying their clinical outcomes. The foregoing should contribute towards reducing the impact of infection and precursor lesions of CC regarding adolescents’ quality of life.
